# Expanding health insurance scheme in the informal sector in Nigeria: awareness as a potential demand-side tool

**DOI:** 10.11604/pamj.2017.27.52.11092

**Published:** 2017-05-19

**Authors:** David Ayobami Adewole, Saidat Abisola Akanbi, Kayode Omoniyi Osungbade, Segun Bello

**Affiliations:** 1 Department of Health Policy & Management, Faculty of Public Health, College of Medicine, University of Ibadan, Nigeria; 2 Department of Epidemiology & Medical Statistics, Faculty of Public Health, College of Medicine, University of Ibadan, Nigeria

**Keywords:** Awareness, health insurance, informal sector, innovative concept, market women, stakeholders

## Abstract

**Introduction:**

The implementation and expansion of a health insurance scheme in the informal sector, particularly in developing countries, is a challenge. With the aid of an innovative Information-Education and Communication model, titled 'Understanding the concept of health insurance: An innovative social marketing tool', an assessment of the awareness and perception of the scheme among market women was carried out.

**Methods:**

This is a cross-sectional descriptive survey, carried out among market women in Ibadan, Nigeria. In a multi-stage sampling technique, a total of 351 women were interviewed using an interviewer-administered, semi-structured questionnaire. The data was analysed using SPSS version 16. Chi-square test was used to test associations between selected variables of interest. Logistic regression model was used to determine predictors of awareness of the National Health Insurance Scheme (NHIS). A model controlling for participants' enrolment status was built and Adjusted Odds Ratio (AOR) reported. Level of statistical significance was set at p < 0.05.

**Results:**

A total of 344 market women aged 18 years and above participated in the study, a response rate of 98.0%. Respondents' educational status was the only predictor significantly associated with awareness of the NHIS. Respondents with post-primary education had 10 times the odds of being aware of the NHIS than respondents with no education or only primary education (Adjusted Odds Ratio = 10.3; 95% CI = 4.1-26.0).

**Conclusion:**

Innovative models to enable potential beneficiaries, especially among the informal sector, to better comprehend and accept the concept of prepayment methods of financing healthcare costs is important in efforts to implement and expand a social health insurance scheme.

## Introduction

Globally, prepayment methods of financing healthcare have been embraced as a viable strategy to achieving universal health coverage [1]. However, it is a relatively new concept in the majority of African countries, including Nigeria [2–4]. State supported social security systems of financing healthcare substantially reduce the burden of healthcare costs on individuals and families. Such social security systems are not common in sub-Saharan African (SSA) countries. The form of social security available in the majority of African countries is the age-old traditional system of reciprocal loans and savings microfinance institutions called Esusu [5], a common financing institution among women's groups [6]. In addition to the fact that the financial bases of these institutions are narrow, financing healthcare is usually not one of the rationales of such traditional systems [7]. Because the majority of the people in the informal sector in low to middle income countries do not have health insurance, despite their willingness to participate in such schemes [6, 8], the alternative choice of payment for health care is the out-of-pocket payment method. Many reasons have been proposed to explain the poor progress of prepayment schemes in Africa [9–11]. Poor engagement in partnerships with the beneficial populations as a result of a poor level of awareness and knowledge of the scheme and its benefits is a major factor [9, 12]. Other factors are poverty, low level of trust in government social policies, and mistrust of fund management in health insurance schemes, while conflicts with religious and cultural beliefs are also common [3, 4, 11]. Electronic media such as radio and television have been cited as sources that could enhance the level of awareness of health insurance [13, 14]. Studies have shown that there is a positive correlation between awareness of and participation in a health insurance scheme. Thus, factors that promote awareness invariably influence participation in the scheme [3, 15, 16]. Other important factors include socio-demographic characteristics such as age, sex, and the marital and educational statuses of intended beneficiaries [17]. It is generally known that individuals and groups of people with a form of formal education are likely to be more responsive to certain policies, especially the health related forms [18, 19]. Potential beneficiaries of health insurance could act as policy 'entrepreneurs'if they are aware and have adequate knowledge of the scheme and its benefits [20]. The National Health Insurance Scheme (NHIS) of Nigeria was established over a decade ago with the sole aim of facilitating access to quality healthcare for all Nigerians. Broadly, there are three different programmes under the scheme designed to ensure that different segments of the populace are catered for. These segments are the Formal and the Informal as well as vulnerable groups such as pregnant women, children under five, prison inmates, retirees and the aged.

Currently, less than 10% of Nigeria's population is beneficiaries of the scheme, and these are mainly in the formal sector. In Nigeria, awareness of prepayment schemes for health is low, as a consequence of inconsistent awareness creation strategies and efforts by the NHIS. As one of the efforts to scale-up the scheme, and to ensure that the informal sector is not disenfranchised, the National Council on Health (NCH) recently approved state ownership of health insurance schemes [21]. The NHIS adopted the family enrolment system whereby a formal sector employee is entitled to enroll a spouse and a maximum of four children under the age of 18 years in addition to himself or herself [12, 22]. It is important that the potential beneficiaries are empowered to play active roles in its design and implementation [10, 23] to encourage its uptake and ensure sustainability. For many reasons associated with reproductive health and related factors, the health seeking behaviour of women is better than that found among men [24]. As the present poor health indicators have established [25], the impact of inadequate access to quality healthcare services in the majority of developing countries is mainly borne by women (and children) [26]. More so, studies have shown that generally compared to men, women are a disadvantaged group in income generating opportunities in Nigeria [27]. Health insurance schemes will enhance women's access to quality healthcare services and they are more likely to support it, compared to men [6]. Empowerment of women through appropriate awareness creation channels will contribute to efforts to promote health insurance schemes among the people. The primary aim of this study was to assess the awareness and perception of market women of prepayment schemes as they are currently available through the NHIS. The secondary aim was to assess determinants of awareness of the prepayment schemes. Findings will contribute to the current efforts in the promotion of prepayment schemes for health, especially in the informal sector and by extension in the general populace.

## Methods

**Study area:** This study was carried out in Aleshinloye market in Ibadan southwest Local Government Area (LGA), Ibadan Oyo State, Nigeria. Ibadan is the capital city of Oyo State, with a population of about 3 million people. Ibadan as a city has a number of institutions of higher learning and research centres, these include the first university in Nigeria - the University of Ibadan, established in 1948 [28]. Nigeria is administratively divided into states, with each state made up of varying numbers of LGAs. Ibadan southwest LGA is one of these, with a population of 283,098 and a total number of 31 healthcare facilities [29]. Within the LGA exists orthodox, alternative and traditional healthcare systems. The orthodox types are majorly the primary and the secondary healthcare providers. The LGAs are mainly responsible for the management of primary healthcare facilities. There was no known health insurance programme for the market women.

This was a cross-sectional, descriptive survey carried out between July 21 and August 29, 2015. The cross-sectional descriptive survey was to obtain the desired representative information on NHIS from the market women at the point of the interview. It involved women traders in Aleshinloye market. The market was chosen among others in Ibadan southwest LGA. It has a population of about 6,000 people of which women make up approximately 90%. The majority of the women trade in goods that include household items, clothing, foodstuffs and similar others. Only market women who were adults 18 years and above, and who owned a stall i.e. physical location in the market, were studied. Market women who worked in the formal sector were excluded. Market women without a specific physical space i.e. without a stall (including hawkers), were not recruited to avoid the difficulty in selecting this category of people during the sampling process. We estimated the sample size using the result of a previous study where the proportion of those who were aware of health insurance in the informal sector was 28.9% [30]. With a power of 80% and a confidence level of 95%, this yielded a sample size of 316, and adjusting for a 10% non-response rate, a total of 351 respondents were recruited for the study. A multi-stage sampling technique was used. Using a simple random sampling technique, 1 out of the 5 LGAs in Ibadan metropolis was selected. In the selected LGA, the largest market was purposively selected. There were two types of traders in the market, those who sell within the space of allocated stalls and those who hawk wares. The sampling frame of the stalls in this market containing 3744 stalls was obtained. Systematic sampling technique was used to select the stalls using a sampling fraction of 1:10. The stall owner or supervisor was enrolled in each of the selected stall.

**Figure 1:**
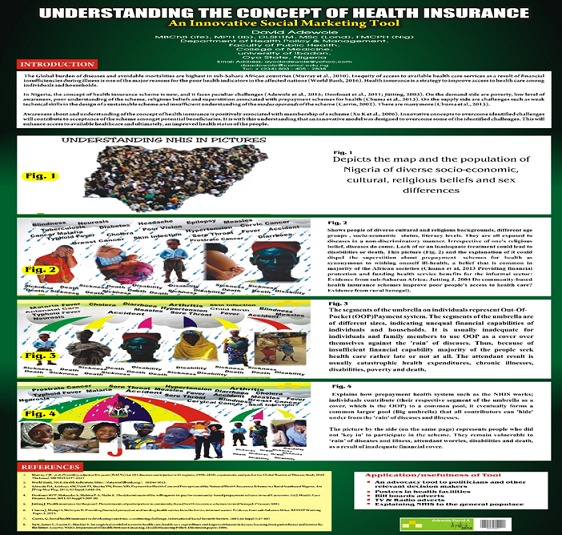
Understanding the concept of health insurance; an innovative social marketing tool

**Data collection:** A semi-structured, pre-tested, interviewer-administered questionnaire was used to collect the data. The questionnaire was adapted from a previous study [**2**]. The questionnaire contained sections on socio-demographic characteristics of respondents, awareness of the availability of health insurance, how health insurance operates, and the attitude of respondents towards health insurance. The tool was pre-tested among market women in Ibadan southeast LGA, about 15km from the study site. Ethical approval to conduct the study was obtained from the University College Hospital Research Ethics Committee in Ibadan, Oyo State. Verbal informed consent was obtained from all participants before the interviews. The basic mechanisms of a prepayment scheme were explained to participants who did not have the knowledge [31]. This explanation was done using a 4-page information-education-communication (IEC) visual aid material titled 'Understanding National Health Insurance (NHIS) in Pictures (Figure 1). The material was designed to enlighten research participants on the basic concepts of fund collection, pooling, purchasing healthcare services and demystifying some superstitious beliefs associated with health insurance schemes in Nigeria and in other similar places. The questionnaire was administered to 1 respondent (a stall owner or the supervisor) in each of the selected stalls. Questions from the respondents about the research were also addressed. Those who declined to participate were excluded from the study.

**Data analysis:** The data was analysed using SPSS version 16. Frequency tables were generated. Chi-square test was used for categorical data to test associations between selected socio-demographic characteristics and awareness of the NHIS, while logistic regression model was used to determine predictors of awareness of the NHIS. Two models were built to investigate predictors of awareness of the NHIS. For both models, the dependent variable was awareness of the NHIS (Yes = 1, No = 0). For the first model, independent predictors of awareness found in previous studies were included in the model. Variables included age (> 40 = 1, ≤= 0), religion (Christianity = 1, Islam = 0), income (> median income of 5000 naira = 1, ≤ median income = 0), marital status (married = 1, single plus others = 0), highest educational attainment (post-primary = 1, primary or no education = 0). In the second model, we tested for the impact of the confounding variable, enrolment in health insurance. Respondents already enrolled were expected to be aware. Level of statistical significance was set at p < 0.05.

## Results

A total of 344 market women aged 18 years and above participated in the study. Thus, the response rate was 98.0%. The mean age of women was 42 ± 12.6 years. As shown in Table 1, people in the age group 40 +/- 41years were in the highest proportion, 29.7%, the majority 80.2% were married, while those who have attained at least secondary school education were 77.6%. Slightly more than two-thirds of the study participants were Christians, 67.7%, while a majority were of the Yoruba ethnic group, 80.5%. More than half, 57.8% claimed to have 3 or more dependents. A great majority, 91.6% used the out-of-pocket (OOP) method to pay healthcare providers. Few of the study participants, 15.1% admitted ever having had difficulties paying for healthcare costs. Of these, almost two-thirds, 30.8% reported borrowing money to source funding during such difficult situations. Others cited other means while a great number, 42.3% did not respond to the question. (Table 2).

**Table 1:** Socio-demographic characteristics of Participants

Socio-demographic Characteristics (N= 344)	Frequency	Percentage
Age		
< 30	53	15.4
30-39	93	27.0
40-49	102	29.7
≥50	96	27.9
Mean Age (std. dev.)	42.52 ±12.6	
**Marital status**		
Single	36	10.5
Married	276	80.2
Others (Separated, divorced, widow, widower)	32	9.3
**Religion**		
Christianity	233	67.7
Islam	111	32.3
**Educational status**		
No formal education	26	7.6
Primary	51	14.8
Secondary	137	39.8
Post-Secondary (Tertiary, Others)	130	37.8
**Ethnicity**		
Yoruba	277	80.5
Igbo	60	17.4
Others	7	2.0
**Number of Dependents (n = 272)**		
0-2	73	21.2
3-5	156	45.3
>5	43	12.5
**Smoking Status**		
Yes	2	0.6
No	342	99.4
**Drinks Alcohol?**		
Yes	2	0.6
No	342	99.4

**Table 2:** Pattern of payment for health care and associated factors, and awareness of health insurance (N = 344)

Variable	Frequency	Percentage
**Method of payment for health care costs**		
Out-of-pocket	315	91.6
Prepayment method	28	8.1
Other forms of payment	1	0.3
**Difficulty paying bills**		
Yes	52	15.1
No	292	84.9
**Hardship payment methods (n = 52)**		
Borrow money	16	30.8
Pay in installments	1	1.9
Gifts/support from family & friends	12	23.1
Others (Sell properties, Use properties as a surety)	1	1.9
No specific answer	22	42.3
**Ever heard about the NHIS**		
Yes	142	41.3
No	202	58.7
**First Source of information about the NHIS (n = 142)**		
Outreach/programs at market *(& Association’s meeting)*	37	26.1
Radio/ TV	47	33.1
a. * *Colleagues *(Friends/Family)*	44	31.0
Hospital	3	2.1
*e.* Others (*Information leaflets/ newspaper* *f. Formal training, others)*	11	7.7
**Health insurance enrolment status (n = 142)**		
Yes	30	21.1
No	108	76.1
No response	4	2.8
**Type of health insurance (n= 30)**		
NHIS	21	70
Private health insurance	4	13.3
No response	5	16.7

**Awareness about NHIS and predictors among respondents:** Just over two-fifths of the total study participants, 41.3% claimed to have ever heard of the NHIS. Electronic media, radio/TV and close associates were mainly the reported first source of information among those who have ever heard about the NHIS in 33.1% and 31.0% respectively. Open campaigns were another significant source of information cited by 26.1% of the people. (Table 3). Of those who claimed to have heard of the NHIS or any form of a prepayment scheme, 21.1% were currently enrolled in any prepayment scheme for health as of the time of the study. Of these, the majority, 70% were enrollees under the NHIS. Generally, respondents were positively disposed towards the NHIS. The majority preferred it to the OOP system as 87.8% were of the opinion that a prepayment scheme as it is available on the NHIS was better. They were also of the opinion that it would minimize financial hardship, 91.3% and would enhance access to good quality healthcare, 90.7 % among others (Table 4). Over two-thirds, 72.1% of the respondents expressed a willingness to participate in the scheme, while 61.0% were skeptical of the scheme. Of this group, expressed concerns centred on unstable income, 33.8%, mistrust with fund management in the scheme, 24.3% and conflicts with religious beliefs, 22.8% among others. Inability to differentiate between the contribution mechanism in health insurance and pre-existing savings and loans arrangement was also cited, 3.8%. Chi square test revealed marital status and educational status as the only significant factors associated with awareness about NHIS. Market women who were single were more likely not to be aware of NHIS (χ^2^ = 14.8, p < 0.001). Market women who had tertiary education were more likely to be aware of NHIS (χ^2^ = 85.3, p <0.001). Only educational status was independently associated with awareness of NHIS in a multivariate analysis. Market women who had post-primary education were ten times more likely to be aware of NHIS than those who had primary or no formal education (Adjusted Odds Ratio = 10.33; 95% CI = 4.12-25.93) (Table 5).

**Table 3:** Respondents’ attitude towards health insurance (N = 344)

Respondents opinion about the NHIS			
Opinion	Agree (%)	Disagree (%)	Don’t Know (%)
It is better than OOP	302(87.8)	34(9.9)	8(2.3)
Minimizes financial hardship	314(91.3)	26(7.6)	4(1.2)
Will encourage others	307(89.2)	30(8.7)	7(2.0)
Enhances access to health care	312(90.7)	27(7.8)	5(1.5)
A good idea	298(86.6)	33(9.6)	13(3.8)
**Respondents Willingness and Expressed Concerns about Participation in a Health Insurance Scheme**			
**Willingness to Participate**	**Frequency**	**Percentage**	
Yes	248	72.1	
No	91	26.5	
No response	5	1.5	
**Reservations about scheme**			
Yes	210	61.0	
No	131	38.1	
No response	3	0.9	
**Expressed Concern About Scheme (n = 210)**			
**Type of concern**			
Conflict with religious beliefs	48	22.8	
Unstable income	71	33.8	
Inability to differentiate scheme from traditional rotational microfinance schemes	8	3.8	
Lack of trust of fund management	51	24.3	
Uncertainty of scheme’s sustainability	32		

**Table 4:** Association between socio-demographic factors and awareness about the NHIS (N = 344)

Variable	Awareness about the NHIS				
	Yes n(%)	No n(%)	Totaln	χ^2^	p-value
**Age**					
<30	20(37.7)	33(62.3)	53	6.9	0.075
30-39	47(50.5)	46(49.5)	93		
40-49	44(43.1)	58(56.9)	102		
>50	31(32.3)	65(67.7)	96		
**Marital Status**					
Single	12(33.3)	24(66.7)	36	14.8	0.001
Married	126(45.8)	149(54.2)	275		
Others^+^	4(12.1)	29(87.9)	33		
**Educational status**					
No formal education/Primary	7(9.1)	70(90.9)	77	85.3	0.000
Secondary	45(31.9)	96(68.1)	141		
Tertiary	90(71.4)	36(28.6)	126		

**Others^+^** Separated, divorced, widow, widower Single and the others in any other type of relationship. More

**Table 5:** Predictors of awareness of the NHIS among market women in Aleshinloye market

Variable	AOR^+^	B^++^	SE^+++^	95% CILower	Upper
Age	0.841	- 0.173	0.284	0.482	1.466
Marital status	0.654	- 0.424	0.407	0.295	1.452
Religion	1.243	- 0.218	0.294	0.699	2.210
Education	10.331	2.335	0.469	4.117	25.926
Tribe	0.812	- 0.208	0.329	0.426	1.548
Income	1.654	- 0.498	0. 627	0.481	5.624

+ AOR = Adjusted Odds Ratio, Hosmer-Lemeshow goodness of fit test: χ ^2^ = 0.473, df = 7, p = 1.0, Model sensitivity = 33.8%, model specificity = 91.1%, (overall prediction accuracy = 67.4%), Cox & Sneil R2 = 0.26, Nagelkerke R2 = 0.34

++ B^++^ = beta

+++ SE^+++^ = standard error Variables: age (> 40 = 1, ≤40 = 0), religion (Christianity = 1, Islam = 0), income (> median income of 5000 naira = 1, ≤ median income = 0), marital status (married = 1, single plus others = 0), highest educational attainment (post-primary = 1, primary or no education = 0)

## Discussion

The response rate in this study was good. The educational status of the study participants was much better than was reported in the most recent National Demographic and Health Survey (NDHS) for Nigeria. However, we recognized the effect of conducting the study in an urban setting with a much higher concentration of better educated people than would have been the case in a rural area. Only marital status and education were significantly associated with being aware of the availability of the NHIS and only education was an independent predictor of awareness. Although age showed a trend at bivariate analysis with the young and the old being less likely to be aware of NHIS, it was not statistically significant and would have been a contrasting finding with previously documented findings. Kirigia et al, (2005) in a study conducted among South African women to determine the ownership of health insurance reported that older people were more likely to buy health insurance because of the tendency to demand health care services as the ageing process sets in [17]. The effect of age on awareness of NHIS may be confounded by education. Exploration of our data showed that older people were significantly less educated and hence after controlling for education, age was no longer associated with awareness of NHIS. Similarly, market women who were married were significantly more educated in our data and after controlling for the effect of education, marital status was also not an independent predictor.

Thus, education appeared to be the single most important consideration responsible for variations in awareness of the NHIS. The reason for those who had higher educational status to be favourably disposed to participating in a health insurance scheme is not unlikely to be associated with the capacity to better comprehend beneficial social policies. In this study, the awareness of health insurance was low. However, among those who were aware of it, a higher education attainment predicted awareness of a health insurance scheme. Educational status may be a factor responsible for the lower awareness in the highest age group. The proportion of those who were aware of the scheme was better among 30 to 49 year-olds than it was among those who were at the extremes of ages. A combined factor of education and marriage could explain this observation as substantiated by previous studies [15, 17]. Because of its multi-pronged benefits, the more educated the people, the more likely they are to have health insurance cover [18, 32]. However, for this to happen, the people must be knowledgeable about the scheme. Foremost in this direction is awareness creation strategies using the most widely available media platforms such as radio and television. Recent successful Ebola disease control in Nigeria and in some other West African countries was partly attributed to effective information dissemination strategies to the general populace [33–35].

Yet, studies have shown that there is currently a low level of awareness of health insurance in developing countries especially among the informal sector of the population [2, 11, 14, 36]. Findings in this study were comparable. Empowerment of the women folk may be one of the most needed strategies to ensure successful health insurance schemes in developing countries. Factors that predispose women to have access to quality health care services for themselves and their wards are most prevalent in SSA [37, 38]. This might enhance the needed motivation for them to serve as policy brokers [20] between state and non-state actors, which oftentimes tends to have conflicting political and economic interests [39–41]. This is more important in the current approach by the Government of Nigeria to implement the State Supported Health Insurance Schemes through the sub-national governments (SSHIS) [21]. Innovative strategies as were used to explain the basic mechanisms of a prepayment scheme to the study participants in this work could be employed. In this study, a few of the women were enrollees under the NHIS. These few got enrolled on the platform of their spouses who were principal enrollees in the formal sector workforce, and who were eligible as stated in the NHIS guideline, to enroll a spouse and four children under the age of 18 years. Expansion of health insurance schemes will enable a shift from the predominant OOP method as shown in this study and previous others [36, 42], and along the continuum towards a much desirable prepayment method for healthcare. This will minimize the predominant poverty predisposing coping strategies such as borrowing to pay for healthcare expenses [43]. The aftermath of borrowing to pay for healthcare expenses could be burdensome but can be minimized greatly through health insurance schemes [44–46]. There is evidence that health insurance could succeed in Nigeria shown by the potential beneficiaries' interests and efforts by stakeholders to implement and expand it. However, interest shown by the people may not necessarily translate into enrolment in the scheme unless expressed areas of concerns are addressed. Conflicts between religious beliefs and the concept of prepayment method for health, lack of trust in government social policies as well as transparency of fund management are some of the issues cited in previous studies that need to be addressed [4, 11].

Similar views were expressed in this study. Another salient issue that came up was the inability of the women to differentiate between premium contributions in a health insurance scheme and the traditional microfinance scheme called Esusu among the Yoruba's in the southwest of Nigeria. Variants of it are also practiced in different parts of Nigeria as well as in other developing countries of Africa and in the Caribbean [47, 48]. Bascom (1952) aptly described it as 'a fund to which a group of individuals make fixed contributions of money at fixed intervals; the total amount contributed by the entire group is assigned to each of the members in rotation' [5]. Although, it is found among small groups of individuals, it is much more common among small scale business owners as a source of business financing and re-financing institutions. As contributions in health insurance are not assigned to contributing members in physical cash as it is the practice in esusu, efforts must be made on the part of the stakeholders to make people understand this important difference. Although the socio-economic status of women in this study was not ascertained, studies have shown a correlation between socio-economic status and participation in a health insurance scheme, and that people of higher socio-economic status were more likely to be in a scheme than were those of lower status [16, 17]. Because women are more likely to be at a disadvantage in income generating activities especially in Nigeria and other developing countries [49], it would be beneficial if the government implemented supportive policies to improve the income generating capacity of women in their businesses as it could translate from a willingness to participate, to an ability to participate in a health insurance scheme [17].

## Conclusion

As stakeholders are making efforts to design and implement health insurance schemes that will incorporate the different strata of the socio-economic groups, there still seems to be no consensus on the best way forward because of the many challenges peculiar to the informal sector group. Stakeholders should not underestimate the support and valuable contributions of communities in the design, implementation and sustainability of health insurance schemes [9, 23]. Successful insurance schemes from many parts of the world especially in Asia and some of the SSA countries have shown the importance of the active involvement of the potential beneficiaries; the government may take the lead role, however, the sustainability of a health insurance policy will depend on the people, accepting and participating in it [9, 10, 23, 32]. Awareness of the NHIS among market women was sub-optimal, awareness creation using innovative concepts to enable the people to comprehend the concept and dispel superstitious beliefs associated with prepayment schemes may aid acceptance. Efforts to promote education may improve awareness of the NHIS, and as such, women's education should be considered a priority and promoted as educated women possess the potential to accept and drive beneficial social policies [6, 18, 32]. The study findings would have been more robust if the socio-economic status of the women had been determined. However, there was no data collected on this. We accept this as a limitation.

### What is known about this topic

- Prepayment schemes such as social health insurance is new in Nigeria and generally awareness about it is low;
- The mechanisms of its operation are largely unknown and the difference between it and the age-old rotational microfinance scheme called Esusu among the Yoruba's is largely unknown;
- The majority of the people ascribe prepayment schemes for health as 'inviting ill-health to oneself'.

### What this study adds

- With the aid of a 4-page innovative Model, this study graphically enlightened market women about the mechanisms of the operation of a prepayment scheme such as is found in a social health insurance scheme ;
- The Model was used to explain a random occurrence of diseases helped in dispelling the superstitious belief associated with prepayment schemes in health ;
- The innovative tool used in this study can be of assistance in the social marketing of a social health insurance scheme to the larger populace.

## Competing interests

The authors declare no competing interest.
